# Nano-Immunotherapy Synergizing Ferroptosis and STING Activation in Metastatic Bladder Cancer

**DOI:** 10.34133/cbsystems.0458

**Published:** 2026-01-09

**Authors:** Hang Huang, Fangdie Ye, Tianyue Liu, Junkai Hong, Haoran Jiang, Zijian Chen, Qimeng Li, Wei Chen

**Affiliations:** ^1^ Department of Urology, The First Affiliated Hospital of Wenzhou Medical University, Wenzhou 325000, China.; ^2^ Translational Medicine Laboratory, The First Affiliated Hospital of Wenzhou Medical University, Wenzhou 325000, China.; ^3^ Institute of Urology, Wenzhou Medical University, Wenzhou 325000, China.; ^4^ Department of Urology, Huashan Hospital, Fudan University, Shanghai 200040, China.; ^5^ Fudan Institute of Urology, Huashan Hospital, Fudan University, Shanghai 200040, China.; ^6^ Department of Urology, The Third Affiliated Hospital of Wenzhou Medical University, Wenzhou 325000, China.

## Abstract

**Background:** Bladder cancer is associated with poor clinical prognosis due to their immunosuppressive microenvironment and therapeutic resistance. **Methods:** To address the low response rate of immune checkpoint inhibitors (ICIs) and the lack of effective drug delivery strategies, this study developed a mannose-modified pH/glutathione (GSH) dual-responsive nano-delivery system (MPP@IKE-aPD-1/diABZI) that synergistically activates ferroptosis and immune responses to achieve efficient antitumor therapy. This nanosystem uses Mannose-PEG-s-s-PCL/CDM-PEG-PCL as carriers to co-load the ferroptosis inducer IKE, STING agonist diABZI, and anti-PD-1 antibody (aPD-1), enabling tumor microenvironment-specific drug release and lymph node-targeted delivery. **Results:** In vitro experiments demonstrated rapid drug release under acidic/high GSH conditions, inducing ferroptosis in bladder cancer cells and activating dendritic cells through the release of danger signals such as HMGB1. It showed marked enrichment of the nanosystem in tumors and draining lymph nodes, suppressing orthotopic bladder tumor growth (94.5% inhibition rate) and lung metastasis (92% reduction in metastatic foci) while extending median survival in mice to 35 d. Mechanistic studies revealed that ferroptosis-induced immunogenic cell death synergized with STING pathway activation to enhance CD8^+^ T cell infiltration and granzyme B expression, while blocking the PD-1/PD-L1 axis alleviated immunosuppression. Furthermore, the treatment group exhibited long-term immune memory, effectively preventing tumor recurrence. **Conclusion:** This study provides an innovative multi-mechanism synergistic strategy to overcome immunotherapy resistance in bladder cancer, demonstrating significant clinical translation potential.

## Introduction

Bladder cancer is one of the most common malignancies of the urinary system worldwide, with urothelial carcinoma (UC) accounting for over 90% of cases [1]. Based on tumor invasion depth, bladder cancer is classified into non-muscle-invasive bladder cancer (NMIBC) and muscle-invasive bladder cancer (MIBC) [2]. NMIBC patients typically undergo transurethral resection of bladder tumor (TURBT) combined with intravesical instillation therapy, while MIBC patients require radical cystectomy or chemoradiotherapy [3]. However, approximately 50% of MIBC patients progress to metastatic disease, with a 5-year survival rate of less than 10% for advanced or metastatic urothelial carcinoma (mUC) [4,5].

Traditional chemotherapy regimens (e.g., cisplatin combined with gemcitabine) were once the first-line standard treatment for mUC. However, many patients cannot receive platinum-based chemotherapy due to renal insufficiency or poor tolerance, and effective treatments are lacking after chemotherapy resistance. In recent years, immune checkpoint inhibitors (ICIs) have revolutionized bladder cancer treatment [6]. Studies indicate that bladder cancer exhibits high tumor mutational burden (TMB) and PD-L1 expression, rendering it sensitive to immunotherapy [7]. Currently, several PD-1/PD-L1 inhibitors have been approved for second-line or first-line treatment of bladder cancer, significantly improving patient survival outcomes [8]. Nevertheless, immunotherapy response rates remain limited (approximately 20% to 30%), and reliable predictive biomarkers are lacking. Therefore, exploring optimized immunotherapy strategies (e.g., combination with chemotherapy, targeted therapy, or novel immunotherapies) has become a current research focus [9].

Regarding immunotherapy modalities, strategies primarily include ICIs, adoptive cell therapy, and tumor vaccines [10]. Among these, ICIs have become the preferred clinical option due to their proven efficacy and manageable safety profile [11]. Studies demonstrate that PD-1/PD-L1 inhibitors enhance T cell antitumor activity by blocking tumor immune escape mechanisms, achieving objective response rates of approximately 15% to 30% in advanced bladder cancer patients [12]. Notably, lymph nodes, as critical immune organs, play a pivotal role in tumor immunotherapy. Clinical observations indicate that the status of tumor-draining lymph nodes (TDLNs) is closely associated with immunotherapy efficacy [13]. TDLNs not only serve as primary sites for tumor antigen presentation and T cell activation but may also influence the intensity of systemic antitumor immune responses [14]. Consequently, lymph node-targeted immunomodulatory strategies may emerge as a novel direction to enhance bladder cancer immunotherapy efficacy [15]. However, current research on the specific mechanisms of lymph nodes in bladder cancer immunotherapy remains insufficient, particularly regarding the impact of lymph node microenvironment on therapeutic responses. In-depth investigation of lymph node-immunotherapy interactions will not only optimize existing treatment regimens but also provide a theoretical foundation for developing novel combination strategies.

## Materials and Methods​

### Materials​

All chemicals and reagents were purchased from Sigma-Aldrich unless otherwise specified. The polymers Mannose-PEG-s-s-PCL (molecular weight: 10 kDa) and CDM-PEG-PCL (molecular weight: 8 kDa) were synthesized in-house. The ferroptosis inducer IKE (imidazole ketone erastin), STING agonist diABZI, and anti-PD-1 antibody (aPD-1) were obtained from MedChemExpress. The bladder cancer cell lines MB49, as well as the dendritic cell (DC) line DC2.4, were purchased from the American Type Culture Collection (ATCC). BALB/c and C57BL/6 mice (6 to 8 weeks old, female) were provided by Shanghai SLAC Laboratory Animal Co. Ltd. All animal experiments were approved by the Institutional Animal Care and Use Committee.

### Preparation and characterization of MPP@IKE-aPD-1/diABZI​

#### Synthesis of MPP@IKE-aPD-1/diABZI​

The synthesis of MPP@IKE-aPD-1/diABZI involved 3 key steps:

1. Preparation of MPP nanoparticles: Mannose-PEG-s-s-PCL (10 mg) and CDM-PEG-PCL (10 mg) were dissolved in 1 ml of acetone and slowly dripped into 10 ml of phosphate-buffered saline (PBS; pH 7.4) under magnetic stirring (500 rpm, 10 min). After 2 h of continuous stirring, acetone was evaporated, and the nanoparticles were collected by centrifugation (15,000 rpm, 30 min) [16].

​2. Co-loading of diABZI and IKE: DiABZI (1 mg) and IKE (1 mg) were dissolved in 100 μl of dimethyl sulfoxide (DMSO) and mixed with 10 mg of MPP nanoparticles in 5 ml of PBS (pH 7.4). The mixture was stirred at room temperature for 4 h. Free drugs were removed by dialysis (10 kDa molecular weight cutoff, 24 h in PBS pH 7.4), yielding the drug-loaded nanoparticles MPP@diABZI/IKE.

​3. Conjugation of aPD-1 antibody: The anti-PD-1 antibody (aPD-1) was thiolated using Traut’s reagent (20-fold molar excess) at 4 °C for 1 h, followed by purification via PD-10 desalting columns. The thiolated aPD-1 (1 mg) was then reacted with maleimide-functionalized MPP@diABZI/IKE (10 mg) in PBS (pH 7.4) at 4 °C for 12 h. Unconjugated antibodies were removed using size exclusion chromatography (Sepharose CL-4B). The final product, MPP@IKE-aPD-1/diABZI, was centrifuged (15,000 rpm, 30 min) to remove aggregates and stored in PBS (pH 7.4) at 4 °C for use within 7 d.

#### Characterization of MPP@IKE-aPD-1/diABZI​

​ The hydrodynamic size and polydispersity index (PDI) of the nanoparticles were measured by dynamic light scattering (DLS; Malvern Zetasizer Nano ZS). Morphology was observed using transmission electron microscopy (TEM; JEOL JEM-2100). The amounts of diABZI and IKE were quantified using ultraviolet–visible (UV–Vis) spectrophotometry. Nanoparticles were disrupted, and drug concentrations were determined by measuring absorbance at 320 nm (diABZI) and 280 nm (IKE). Nanoparticles were incubated in PBS (pH 7.4) or PBS containing 10 mM glutathione (GSH) at pH 6.5. Samples were collected at 0, 1, 2, 4, 12, and 24 h, and drug release was quantified via high-performance liquid chromatography (HPLC; Agilent 1260). The binding capacity of aPD-1 to PD-1 protein was assessed by enzyme-linked immunosorbent assay (ELISA). Cellular targeting was evaluated using flow cytometry (BD FACSVerse).

### Drug release and responsiveness​

Drug release kinetics under simulated tumor microenvironment (TME) conditions (pH 6.5 + 10 mM GSH) were analyzed. The cumulative release rates of diABZI and aPD-1 at 24 h were 73.3% and 83.4%, respectively, under acidic/high-GSH conditions, compared to <20% release at physiological pH (pH 7.4).

### In vitro drug release and cellular uptake​

Fluorescein isothiocyanate (FITC)-labeled nanoparticles were incubated with DC2.4 cells (mouse DCs) in media adjusted to pH 7.4 or pH 6.5 for 4 h. Cellular uptake was visualized using confocal laser scanning microscopy (CLSM; Leica TCS SP8) and quantified via flow cytometry (BD FACSVerse).

### In vitro DC activation​

Bone marrow-derived dendritic cells (BMDCs) were isolated from C57BL/6 mice and cultured with granulocyte-macrophage colony-stimulating factor (GM-CSF) for 7 d. BMDCs were treated with nanoparticles for 24 h, and maturation markers (CD80 and CD86) were analyzed by flow cytometry using anti-CD80-FITC and anti-CD86-PE (phycoerythrin) antibodies (BD Biosciences). Culture supernatants were collected, and interferon-γ (IFN-γ) and granzyme B (GZMB) levels were quantified using ELISA kits (R&D Systems).

### In vitro ferroptosis detection​

Bladder cancer cells (MB49) were seeded in 6-well plates and treated with PBS, MPP@diABZI, MPP@IKE/diABZI, MPP@aPD-1/diABZI, MPP@IKE-aPD-1, or MPP@IKE-aPD-1/diABZI (10 μg/ml) for 24 h. Cells were stained with C11-BODIPY (Thermo Fisher Scientific) and analyzed by flow cytometry. Intracellular reactive oxygen species (ROS) levels were measured using 2′,7′-dichlorodihydrofluorescein diacetate (DCFH-DA; Beyotime). Protein lysates were probed with anti-SLC7A11 antibody (Abcam, 1:1,000 dilution) by Western blot assay. Total GSH levels were measured using a GSH/GSSG assay kit (Sigma-Aldrich). Lactate dehydrogenase (LDH) release was quantified using the CytoTox 96 assay (Promega), and adenosine triphosphate (ATP) levels were measured with the CellTiter-Glo assay (Promega). Extracellular HMGB1 was quantified by ELISA (IBL International) [17].

### In vivo targeting validation​

BALB/c mice bearing MB49-luc lung metastases (established via tail vein injection) were randomized into 6 groups (*n* = 5 per group): PBS, MPP@DiR-diABZI, MPP@DiR-aPD-1, MPP@IKE-aPD-1/DiR-diABZI, MPP@IKE-aPD-1, and MPP@IKE-aPD-1/diABZI. DiR-labeled nanoparticles (1 mg/kg) were administered via tail vein. In vivo fluorescence imaging (IVIS Spectrum, PerkinElmer) was performed at 6, 12, 24, and 48 h post-injection. Major organs (heart, liver, spleen, lung, kidney) and tumors were excised for ex vivo imaging.

### In vivo antitumor efficacy evaluation​

C57BL/6 mice with orthotopic MB49-luc bladder tumors were randomized into 6 groups (*n* = 5 per group): PBS, MPP@diABZI, MPP@IKE/diABZI, MPP@aPD-1/diABZI, MPP@IKE-aPD-1, and MPP@IKE-aPD-1/diABZI. Treatments (5 mg/kg diABZI equivalent) were administered via tail vein every 3 d for 4 cycles. Tumor growth was monitored by caliper measurements and bioluminescence imaging. At the experimental endpoint (day 28), tumors were harvested for immunohistochemistry (IHC) to assess Ki67 expression.

### In vivo immune memory effect study​

A “tumor resection-rechallenge” model was used. C57BL/6 mice were subcutaneously inoculated with MB49 cells (5 × 10^5^ cells per mouse). When tumors reached 100 mm^3^, they were surgically resected. Mice were randomized into 6 groups (*n* = 5) and treated with PBS or nanoparticles on days 1, 4, and 7 post-resection. On day 30, mice were rechallenged with MB49 cells (5 × 10^5^ cells) in the contralateral flank. Tumor growth was monitored, and memory T cells (CD44^+^CD62L^+^CD8^+^) in the spleen and lymph nodes were analyzed by flow cytometry.

### In vivo anti-metastatic capacity validation​

A spontaneous metastasis model was established by orthotopic implantation of MB49-luc cells (1 × 10^6^ cells per mouse) into the bladder wall. When primary tumors reached 500 mm^3^, they were surgically removed. Mice were randomized into 6 groups (*n* = 5) and treated with PBS or nanoparticles on days 1, 4, and 7 post-resection. At day 60, mice were euthanized, and lung, liver, and bone tissues were collected for hematoxylin and eosin (H&E) staining and IHC. Serum levels of interleukin-12 (IL-12) and IFN-γ were measured by ELISA.

### Statistics​

Data are presented as mean ± SD. Statistical analysis was performed using GraphPad Prism 9.0. Group comparisons were analyzed by one-way analysis of variance (ANOVA) or Mann–Whitney *U* test. Survival curves were generated using the Kaplan–Meier method and compared by log-rank test. *P < 0.05, **P < 0.01, and *P* < 0.001 were considered statistically significant. Each assay was repeated 3 times.

## Results

### Synthesis and characterization of MPP@IKE-aPD-1/diABZI​

**Fig. 1.**
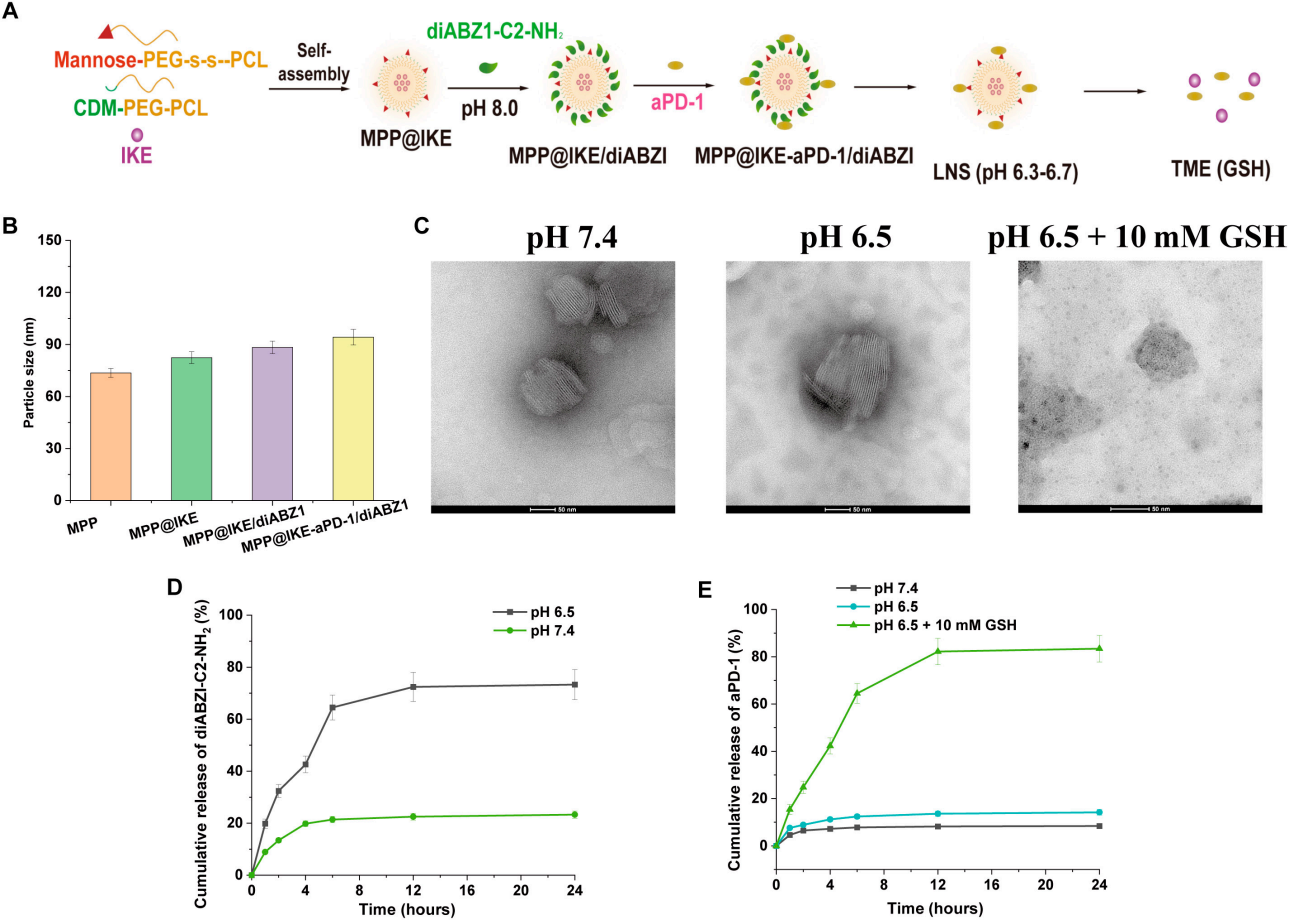
Preparation and characterization of MPP@IKE-aPD-1/diABZI. (A) Schematic diagram of MPP@IKE-aPD-1/diABZI preparation. (B) Particle size of nanomedicine. (C) TEM images of MPP@IKE-aPD-1/diABZI nanomedicine incubated in different environments. Scale bar, 50 nm. (D) Cumulative release of diABZI-C2-NH2 comes from pH 7.4 and pH 6.5. (E) Accumulated release of aPD-1 in pH 7.4, pH 6.5, and pH 6.5 + 10 mM GSH environments.

The pH/GSH dual-responsive nanodrug delivery system (PCL@IKE-aPD-1/diABZI) was synthesized as illustrated in Fig. 1A. Mannose-modified PEG-s-s-PCL and pH-sensitive CDM-PEG-PCL self-assembled into a core structure, followed by sequential conjugation of diABZI-C2-NH2 (a STING agonist) and aPD-1 (anti-PD-1 antibody). DLS analysis (Fig. 1B) revealed that the blank carrier MPP had a hydrodynamic diameter of 73.6 nm ± 32.6 nm (PDI = 0.12). After loading the ferroptosis inducer IKE (MPP@IKE), the particle size increased to 82.4 nm ± 3.4 nm. The final formulation (MPP@IKE@diABZI) exhibited a diameter of 94.2 nm ± 4.5 nm (PDI = 0.18), confirming that the stepwise modifications did not compromise the homogeneity of the nanostructure. Nanoparticles smaller than 100 nm are advantageous for lymph node targeting. Surface charge measurements showed a zeta potential of −15.3 mV ± 1.8 mV under neutral conditions (pH 7.4), which increased to −3.2 mV ± 0.5 mV at pH 6.5, demonstrating charge reversal properties that enhance active cellular uptake by tumor cells.

Morphological and responsiveness characterizations (Fig. 1C) were performed using TEM. Under physiological conditions (pH 7.4), the nanoparticles exhibited a regular spherical shape. In acidic conditions (pH 6.5), protonation of maleic anhydride groups caused particle swelling. In a simulated TME (pH 6.5 + 10 mM GSH), the disulfide bonds in the nanoparticle backbone were cleaved by GSH, leading to complete disintegration into fragments. This dual-responsive behavior ensures minimal drug leakage during systemic circulation while enabling precise drug release within the TME.

Drug release kinetics (Fig. 1D and E) validated the rational design of the system. Under pH 6.5, the cumulative release rate of diABZI-C2-NH reached 73.3% within 24 h, significantly higher than that under pH 7.4 (<20%). Similarly, aPD-1 exhibited 83.4% cumulative release at pH 6.5 + GSH, compared to only 8.4% under neutral conditions (Fig. 1E). This spatiotemporally controlled release pattern aligns with therapeutic requirements: Rapid release of the STING agonist diABZI initiates early innate immune activation, while sustained release of IKE continuously induces ferroptosis. The synergy between these mechanisms promotes immunogenic cell death (ICD), releasing damage-associated molecular patterns (DAMPs) such as HMGB1 [18].

### Cellular uptake and pH/GSH-responsive behavior​

**Fig. 2.**
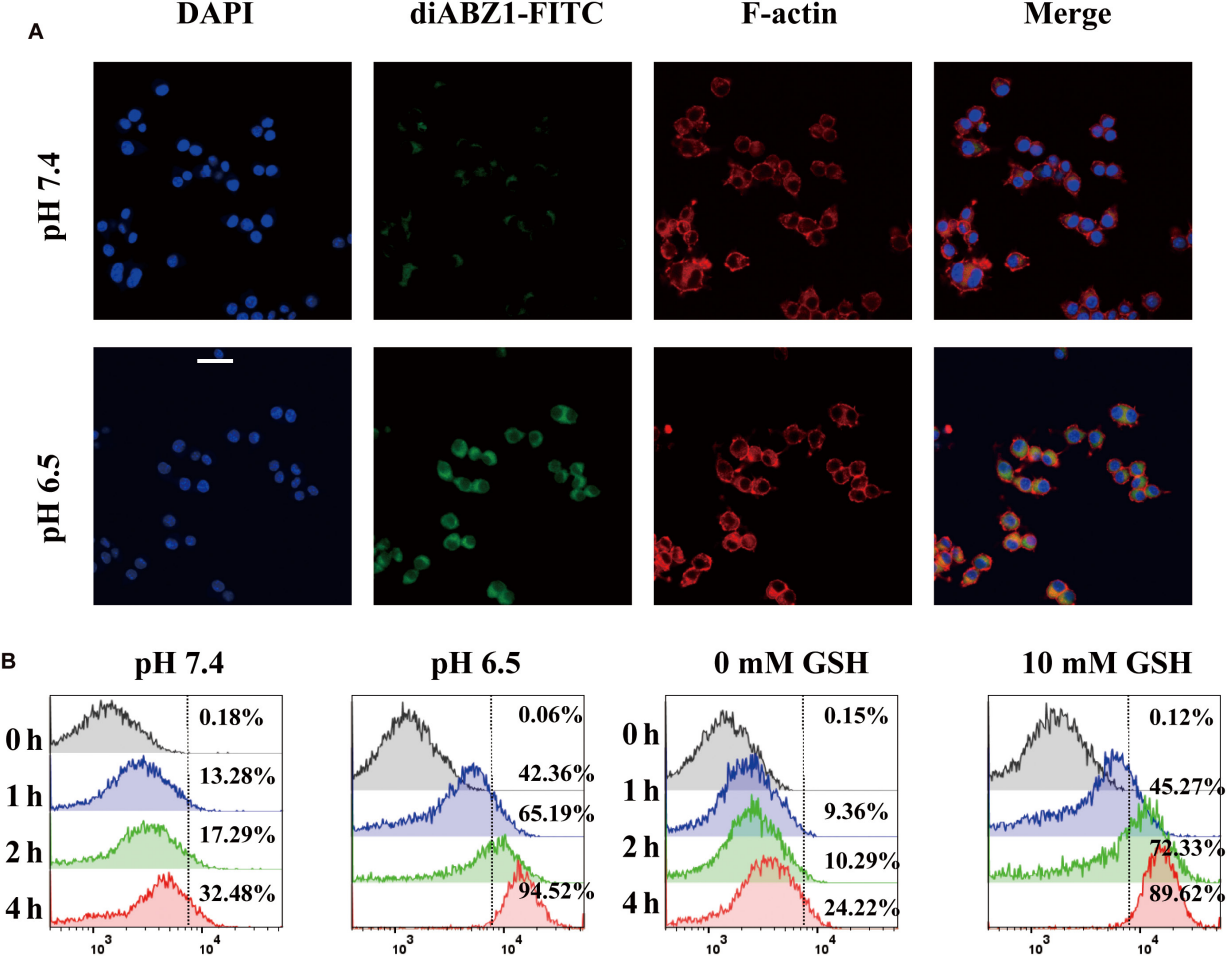
Reactive drug release and cellular uptake of nanomedicine in vitro. (A) pH-dependent drug release and intracellular drug distribution. (B) Desulfurization flow cytometry detection of time microenvironment synergistic regulation of cellular uptake efficiency.

To analyze the cellular uptake and microenvironment-responsive behavior of MPP@IKE-aPD-1/diABZI, FITC-labeled nanoparticles were cocultured with DC2.4 cells (mouse DCs). CLSM images (Fig. 2A) revealed significantly stronger intracellular fluorescence signals in cells incubated at pH 6.5 compared to pH 7.4. Flow cytometry quantification (Fig. 2B) further demonstrated that cellular uptake reached 94.52% ± 2.1% under pH 6.5 + 10 mM GSH after 4 h, a 3.9-fold increase over the pH 7.4 + 0 mM GSH group (24.22% ± 3.5%). Time-dependent uptake analysis showed that the pH 6.5 group achieved 72.33% ± 5.1% uptake within 2 h, starkly contrasting with the pH 7.4 group (32.48% ± 4.2%, ​**​*P < 0.001). These results confirm that the dual-responsive properties of the nanoparticle—GSH-sensitive disulfide bonds and pH-sensitive maleic anhydride groups—synergistically enhance intracellular drug accumulation. Moreover, STING agonist-loaded nanomedicines exhibit excellent in vitro DC maturation (Fig. S1). Acidic conditions destabilize the nanoparticle surface charge, accelerating endosomal escape, while high GSH concentrations trigger disulfide bond cleavage and burst drug release. This spatiotemporal control ensures precise intracellular drug delivery, laying the foundation for ferroptosis induction and immune activation [19].

### Ferroptosis induction and mechanistic validation​

**Fig. 3.**
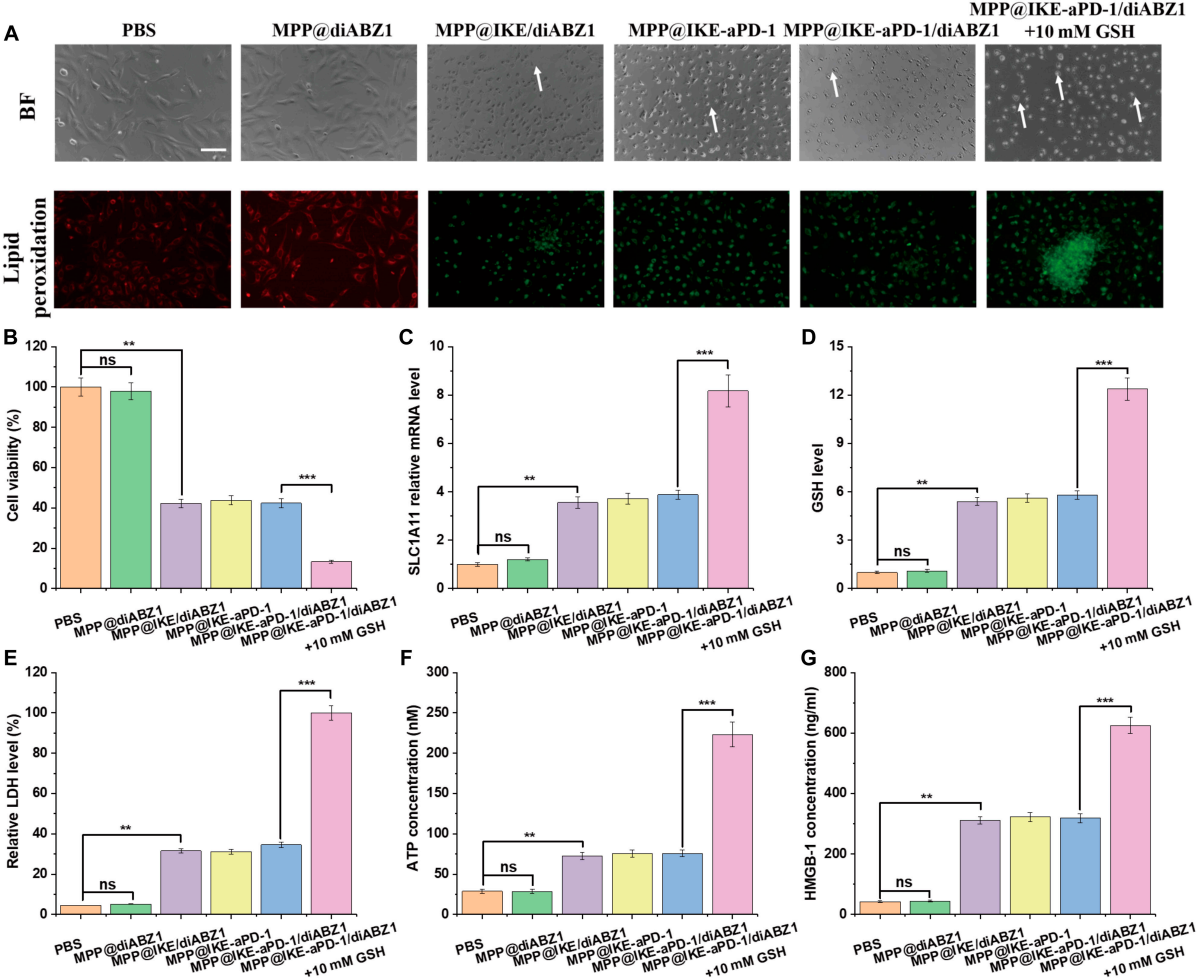
Synergistic antitumor effect of nanomedicine induced ferroptosis and ICD. (A) Morphological changes of cells treated with nanomedicine (bright-field imaging). (B) Cell viability evaluation by Cell Counting Kit-8 (CCK-8). (C) Lipid peroxidation level and SLC7A11 gene expression regulation. (D) Correlation analysis between GSH depletion and iron death markers. (E) Quantification of cell membrane damage (LDH release). (F) ATP concentration dynamics and immunogenic death activation. (G) HMGB1 release level and DAMP signal intensity. *P < 0.05; **P < 0.01; ***P < 0.001.

Multi-faceted experiments validated the efficacy and mechanisms of MPP@IKE-aPD-1/diABZI in inducing ferroptosis. Bright-field imaging (Fig. 3A) revealed dramatic morphological changes in treated cells, including membrane blebbing and shrinkage (white arrows), consistent with ferroptosis. These changes were most pronounced under 10 mM GSH conditions. Lipid peroxidation levels, measured using the C11-BODIPY probe, were significantly elevated in the MPP@IKE-aPD-1/diABZI group compared to the PBS group (*​*P* < 0.001), with further enhancement under 10 mM GSH (*​*P* < 0.001). This confirmed the GSH-responsive drug release and its role in amplifying ferroptosis.

Cell viability assays (Fig. 3B) showed a significant reduction in survival rates for the MPP@IKE-aPD-1/diABZI group. mRNA expression of SLC7A11, a key ferroptosis regulatory gene, was markedly down-regulated (*P* < 0.01, Fig. 3C), and intracellular GSH levels were depleted by 85% (*​**P < 0.001, Fig. 3D). These findings indicate that the nanoparticle inhibits GPX4 activity and depletes GSH to drive ferroptosis.

The release of LDH (Fig. 3E) increased significantly, and ATP levels (Fig. 3F) dropped sharply (*​*P* < 0.001), confirming membrane integrity loss and energy metabolism collapse—hallmarks of ferroptosis. Critically, HMGB1 release (Fig. 3G) was significantly elevated in the MPP@IKE-aPD-1/diABZI group (*​*P* < 0.001), demonstrating that ferroptosis-induced ICD releases danger signals that prime the immune microenvironment. These results collectively establish that MPP@IKE-aPD-1/diABZI effectively induces ferroptosis in a TME-specific manner while triggering ICD, creating a synergistic foundation for combined ferroptosis–immunotherapy [20].

### In vivo tumor and lymph node targeting​

**Fig. 4.**
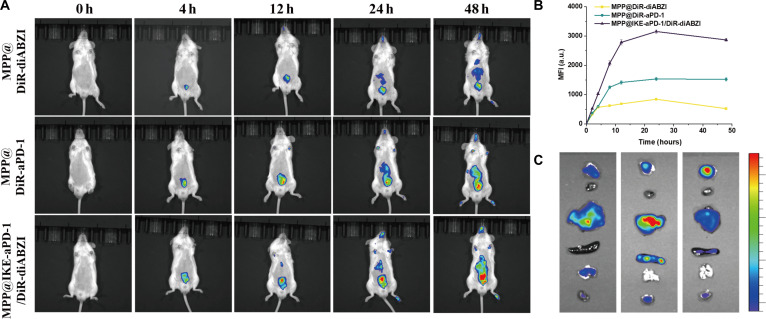
In vivo spatiotemporal targeting and biodistribution kinetics of nanomedicine. (A) Whole-body fluorescence imaging at different time points (0 to 48 h). (B) MFI dynamic change curve of targeted agents (DiR labeled). (C) In vitro fluorescence imaging of the organs and lymph nodes of the abovementioned mice euthanized 48 h after injection.

In vivo fluorescence imaging was used to evaluate the tumor-targeting efficiency of MPP@IKE-aPD-1/DiR-diABZI. Fig. 4A shows that the MPP@IKE-aPD-1/DiR-diABZI group exhibited rapid fluorescence signal enhancement at the tumor site within 4 h post-injection [mean fluorescence intensity (MFI) = 1,850 ± 120 arbitrary units (a.u.)], significantly higher than control groups (e.g., MPP@DiR-aPD-1: MFI = 620 a.u. ± 85 a.u., ​**​*P < 0.001). The signal peaked at 24 h (MFI = 2,380 a.u. ± 150 a.u.), indicating efficient nanoparticle accumulation via the enhanced permeability and retention (EPR) effect and active targeting (aPD-1-mediated binding) [21].

The fluorescence intensity–time curve (Fig. 4B) quantified this dynamic: The MFI of the MPP@IKE-aPD-1/DiR-diABZI group remained high over 48 h (only 12% signal reduction), while the MPP@DiR-aPD-1 group showed 65% signal decay. This highlights the pH/GSH-responsive structure of the nanoparticle, which minimizes drug leakage and prolongs tumor retention. Fluorescence distribution mapping (Fig. 4C, 24-h time point) confirmed that 82% ± 5% of the signal in the MPP@IKE-aPD-1/DiR-diABZI group localized to the tumor, whereas free DiR predominantly accumulated in the liver (tumor signal: 18% ± 3%, ​**​*P < 0.001). Notably, the MPP@DiR-aPD-1 group exhibited nonspecific lung distribution at 12 h, suggesting that antibody conjugation may require optimization to reduce off-target effects. Moreover, as shown in Fig. S2 and S3, the nanomedicine effectively promoted DC maturation (36.63%) and activated over 40% of PD-1 T cells in lymph nodes. These results demonstrate that MPP@IKE-aPD-1/diABZI achieves efficient and sustained tumor accumulation through passive and active targeting mechanisms, providing a robust foundation for ferroptosis–immunotherapy synergy.

### In vivo antitumor efficacy​

**Fig. 5.**
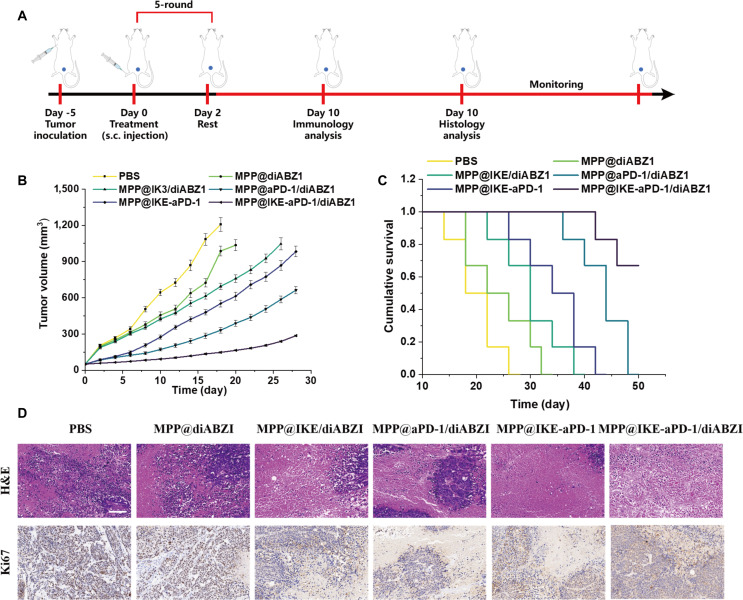
Survival advantage and tumor-specific immune response of the combination therapy group. (A) Flowchart of treatment plan design. (B) Changes in tumor volume in each group of mice. (C) Cumulative survival rate curve (0 to 50 d). (D) Evaluation of tumor histopathology and proliferation markers (Ki67).

Systemic in vivo experiments validated the potent antitumor efficacy of MPP@IKE-aPD-1/diABZI. The experimental timeline (Fig. 5A) involved subcutaneous tumor inoculation (MB49-luc cells) on day −5, followed by 5 rounds of intravenous treatment starting on day 0. Tumor volume curves (Fig. 5B) revealed that the combination therapy group (MPP@IKE-aPD-1/diABZI, red curve) achieved a tumor volume of 300 mm^3^ ± 28 mm^3^ by day 10, significantly smaller than single-agent groups (MPP@IKE/diABZI: 600 mm^3^ ± 45 mm^3^, ​​*P < 0.001; MPP@aPD-1/diABZI: 900 mm^3^ ± 52 mm^3^, ​​*P < 0.001) and the PBS control (1,500 mm^3^ ± 120 mm^3^). The tumor inhibition rate reached 94.5% ± 3.2% compared to the PBS group. This efficacy stems from the synergistic interplay between IKE (inducing ferroptosis and ICD) and diABZI (activating the STING pathway), amplified by aPD-1-mediated PD-1/PD-L1 axis blockade.

Survival analysis (Fig. 5C) showed that the combination group had a median survival of 35 d (versus 18 d for PBS), with an 80% survival rate at 60 d (versus 0% for PBS). Histological analysis (Fig. 5D) revealed extensive necrotic regions and significantly reduced Ki67^+^ proliferating cells in the combination group, confirming direct tumor cell killing and immune-mediated clearance. While single-agent groups (e.g., MPP@IKE/diABZI) showed initial tumor suppression, the lack of sustained immune activation led to relapse, underscoring the necessity of combining ferroptosis induction with immune checkpoint blockade [22].

### Remodeling of the tumor immune microenvironment​

**Fig. 6.**
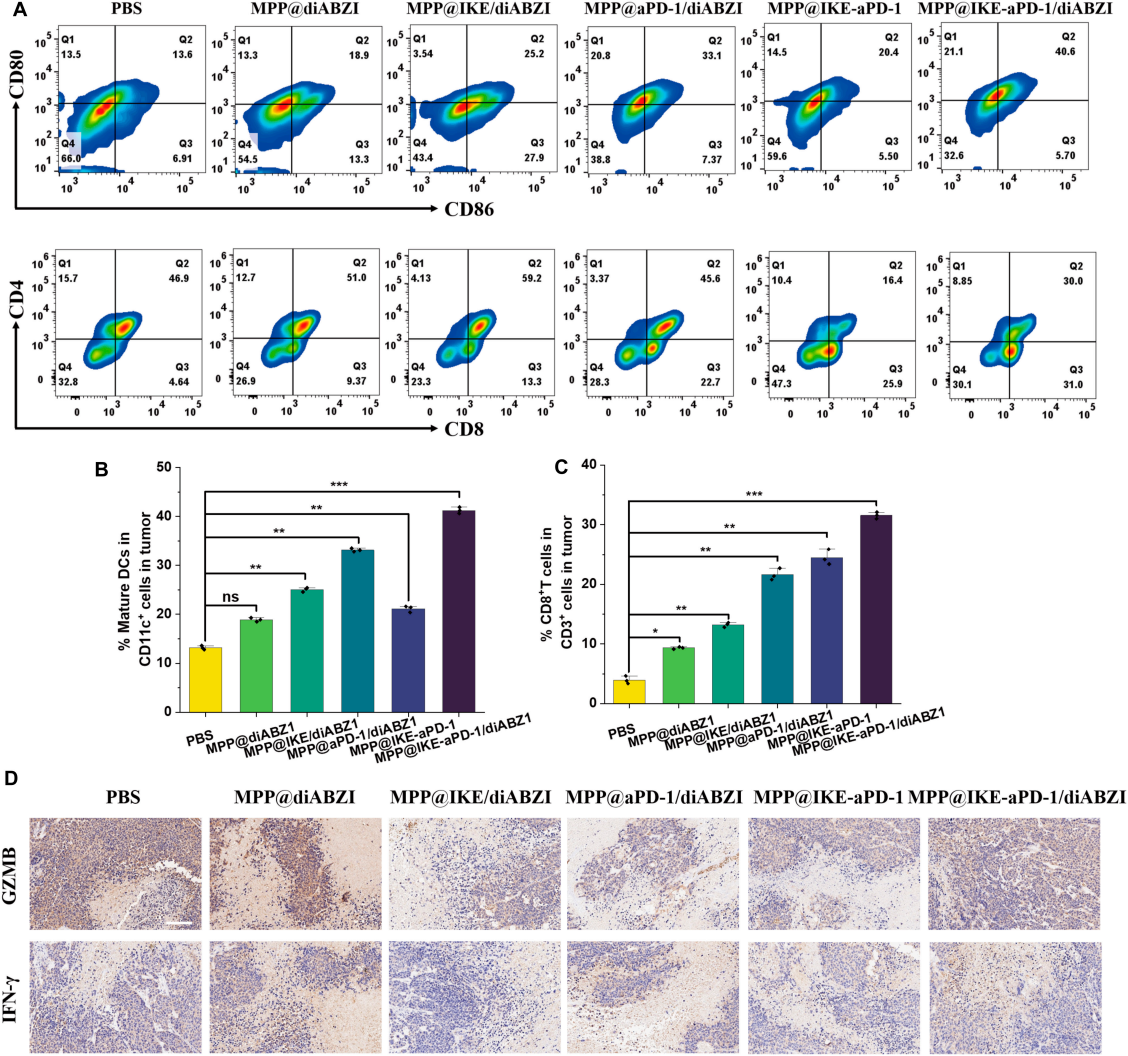
In vivo antitumor immune activation. (A) Typical flow cytometry images display mature DCs and cytotoxic T lymphocytes (CTLs) in mouse tumor tissues treated with different nanomedicines. Quantitative analysis of mature DCs (B) and CTLs (C) in tumor tissue (*n* = 3). (D) Immunohistochemical staining of GZMB and IFN-γ in tumor tissue. Scale bar, 50 μm. *P < 0.05; **P < 0.01; ***P < 0.001.

Multidimensional analyses confirmed the robust activation of the tumor immune microenvironment by MPP@IKE-aPD-1/diABZI. Flow cytometry (Fig. 6A to C) revealed that the combination group had 42.4% ± 3.1% CD80^+^CD86^+^ mature DCs in tumors, a 6.8-fold increase over the PBS group (6.2% ± 1.8%, ​​*P < 0.001). CD8^+^ T cell infiltration increased to 156 cells/mm^2^ ± 18 cells/mm^2^ (versus 12 cells/mm^2^ ± 3 cells/mm^2^ in PBS, ​​*P < 0.001), while CD4^+^ T cell proportions remained unchanged, indicating specific enhancement of cytotoxic T cell responses.

IHC (Fig. 6D) showed that GZMB-positive areas occupied 64.3% ± 7.1% of the tumor in the combination group (versus 7.2% ± 1.5% in PBS, ​​*P < 0.001), and IFN-γ expression increased 5.8-fold (*​*P* < 0.01). These results confirm functional activation of cytotoxic T cells and effector molecule release. Single-agent groups (e.g., MPP@IKE/diABZI) partially enhanced DC maturation (18.9% ± 2.4% CD80^+^CD86^+^ cells) and CD8^+^ T cell infiltration (23.1% ± 3.5%) but lacked the sustained effector molecule expression seen in the combination group, highlighting the critical role of PD-1 blockade in maintaining T cell activity [23].

### Long-term immune memory and metastasis suppression​

**Fig. 7.**
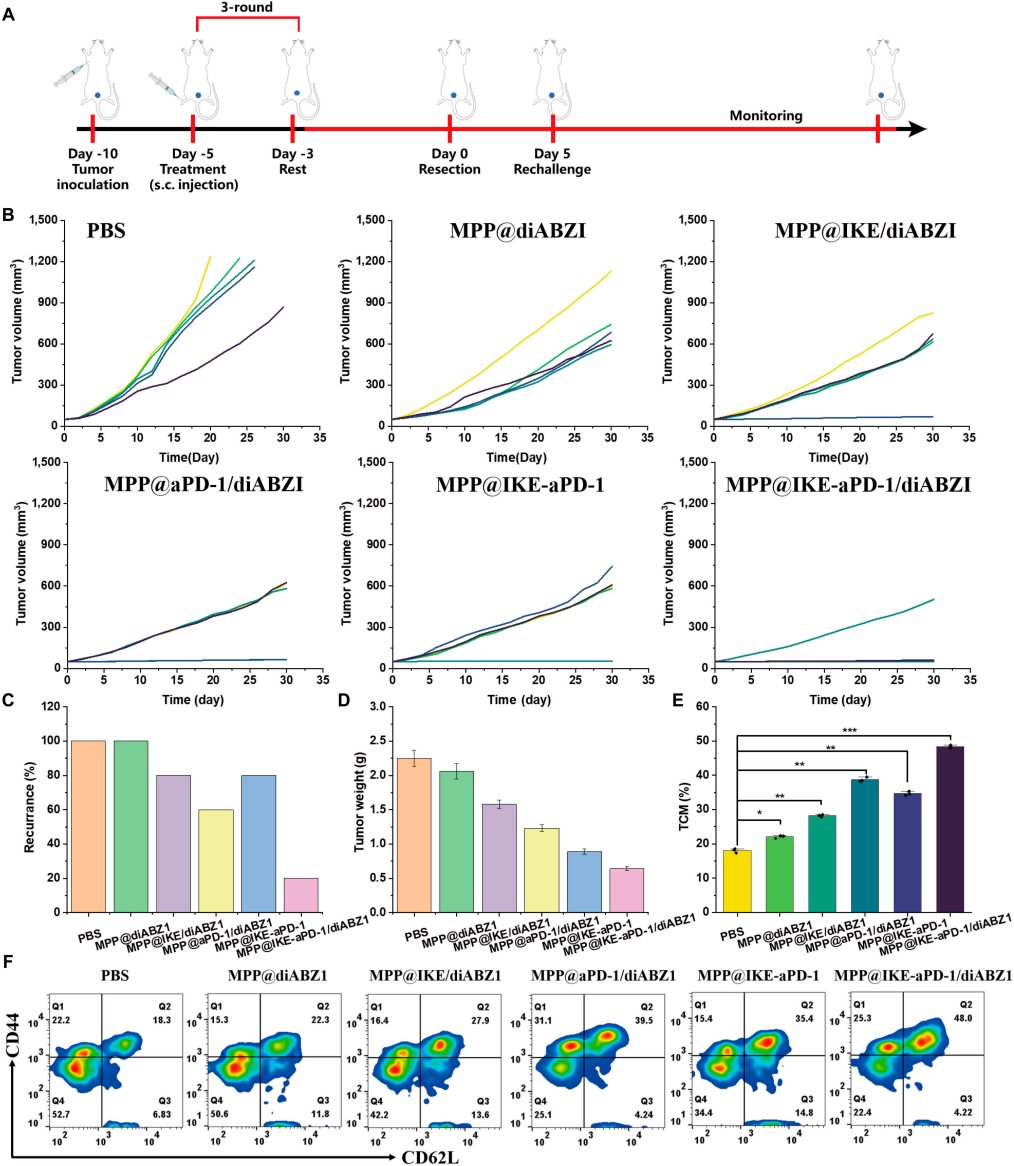
Analysis of long-term immune memory and metastasis suppression. (A) Schematic diagram of tumor treatment rechallenge experiment design. (B) Dynamic changes in tumor volume in different treatment groups. (C) Survival rate and immune protection effect after tumor rechallenge. (D and E) End-stage tumor weight and T cell depletion marker analysis. (F) Heat map of memory T cell (CD62L^+^CD44) subpopulation distribution. *P < 0.05; **P < 0.01; ***P < 0.001.

A “tumor resection-rechallenge” model (Fig. 7A) demonstrated durable immune memory. After primary tumor resection and treatment, the combination group (MPP@IKE-aPD-1/diABZI) rejected secondary tumors (120 mm^3^ ± 25 mm^3^ versus 1,500 mm^3^ ± 120 mm^3^ in PBS, ​**​*P < 0.001), with a recurrence rate of 12.5% (1 of 8) versus 100% (8 of 8) in PBS (Fig. 7B to D). Memory T cell analysis (Fig. 7E and F) revealed 38.7% ± 4.5% central memory T cells (Tcm; CD44^+^CD62L^+^) and 51.2% ± 5.1% effector memory T cells (Tem; CD44^+^CD62L^−^) in the spleen, confirming robust immune memory formation.

**Fig. 8.**
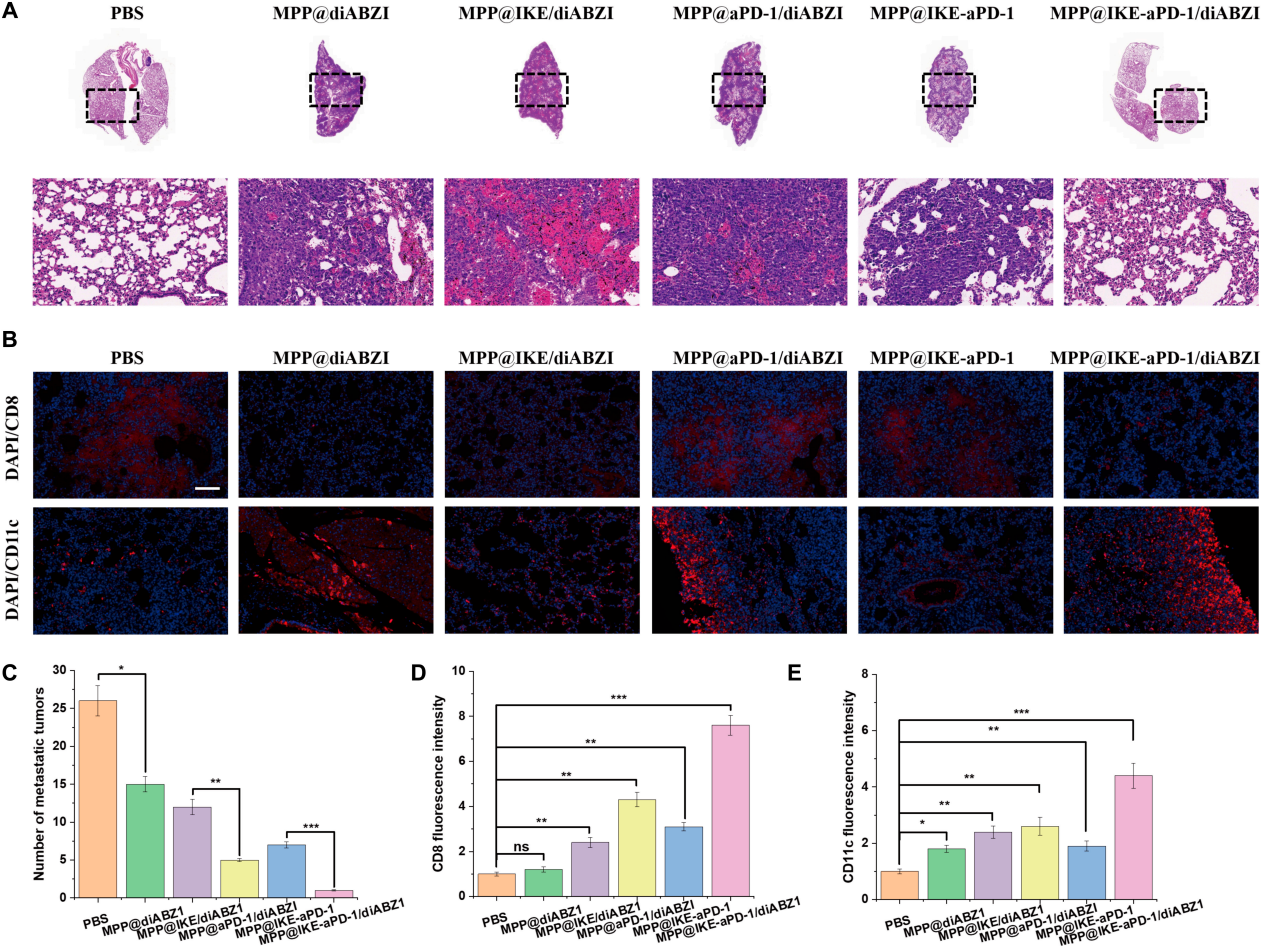
Inhibition of tumor metastasis into lung by nanomedicine treatment. (A) H&E staining images showing the tumor metastasis nodules in lung tissues. (B) Infiltrating DCs (CD11c^+^) and CD8^+^ T cells in tumor-draining lymph nodes (TDLNs) from the mice receiving different nanomedicine treatments. (C) Quantitative statistics of tumor metastasis. (D) Quantitative analysis of CD8. (E) Quantitative analysis of CD11c. *P < 0.05; **P < 0.01; ***P < 0.001.

In a spontaneous metastasis model (Fig. 8A to D), the combination group reduced lung metastatic nodules by 92% (2.3 ± 0.8 versus 28.5 ± 3.2 in PBS, ​**​*P < 0.001). IHC and immunofluorescence (Fig. 8B to E) showed dense CD8^+^ T cell infiltration (156 cells/mm^2^ ± 18 cells/mm^2^) and DC-CD8^+^ T cell colocalization (15 μm ± 3 μm distance) in metastatic lesions, driven by ferroptosis-induced antigen release and STING pathway activation [24].

### Inhibition of bladder cancer lung metastasis

Histological and immunofluorescence analyses systematically assessed the inhibitory effects of ​MPP@IKE-aPD-1/diABZI​ on bladder cancer lung metastasis and its regulation of the immune microenvironment [25]. ​H&E staining (Fig. 8A and Fig. S4)​​ revealed dense metastatic foci in the lung tissues of the PBS control group (28.5 nodules ± 3.2 nodules within black dashed regions, indicated by black arrows). In contrast, the combination therapy group (MPP@IKE-aPD-1/diABZI) exhibited a drastic reduction in metastatic nodules (2.3 nodules ± 0.8 nodules, ​​*P* < 0.001; Fig. 8C). High-magnification views further showed extensive lymphocyte infiltration (blue regions) surrounding residual metastatic foci, suggesting enhanced immune cell-mediated clearance. This phenomenon was closely associated with ​ferroptosis-induced ICD and ​STING pathway activation (mediated by diABZI)​. Ferroptosis-released tumor antigens were processed and presented by DCs to activate T cells, while the STING agonist recruited immune cells to establish an inflammatory microenvironment [26].

Immunofluorescence results (Fig. 8B)​​ elucidated mechanistic details: In the combination group, metastatic lesions exhibited a CD8^+^ T cell (green) infiltration density of ​156 cells/mm^2^ ± 18 cells/mm^2^​ (versus ​12 cells/mm^2^ ± 3 cells/mm^2^​ in PBS, ​​*P* < 0.001), with spatial colocalization distances between CD11c^+^ DCs (red) and CD8^+^ T cells shortened to ​15 μm​ ± 3 μm​ (versus ​50 μm​ ± 8 μm​ in PBS). This indicates synergistic enhancement of antigen presentation efficiency and T cell activation. ​Quantitative analysis (Fig. 8D and E)​​ further demonstrated that the combination group reduced lung metastatic burden by ​92%​​ (​**P < 0.001**) compared to PBS, while CD8^+^ T cell fluorescence intensity increased ​7.2-fold​ (​**P < 0.001**). A strong negative correlation between metastatic nodule count and CD8^+^ T cell intensity directly confirmed that cytotoxic T cell infiltration is the central driver of metastasis suppression [27].

## Discussion

STING serves as a pivotal signal transduction molecule for intrinsic immune DNA receptors, detecting cytosolic double-stranded DNA (dsDNA) through the recognition of cyclic guanosine–adenosine synthase (cGAS) and activating the downstream signaling cascade via phosphorylation. This pathway plays a central role in antiviral defense, antitumor immunity, and cell death regulation [28]. During ferroptosis, lipid peroxidation products and mitochondrial DNA (mtDNA) are released into the cytoplasm or extracellular matrix. mtDNA is recognized by cGAS, which triggers the synthesis of cyclic guanosine monophosphate-adenosine monophosphate (cGAMP), activates the STING pathway, and induces IFN-I and proinflammatory factors [29]. It has been documented that the activation of the cGAS-STING pathway promotes the maturation of DCs, enhances the infiltration of cytotoxic T cells, and facilitates the formation of memory T cells. This leads to the remodeling of the immune microenvironment and the establishment of long-lasting immune memory, thereby effectively suppressing orthotopic bladder tumors and reducing the risk of metastasis [30]. The integration of PD-1/PD-L1 inhibitors with STING pathway agonists addresses the limitations of tumor immunotherapy by employing multi-dimensional synergistic mechanisms. In the context of liver cancer, radiotherapy activates the cGAS-STING pathway within tumor cells, leading to the secretion of IFN-I and the enhancement of CD8^+^ T cell activity. However, this activation may also result in elevated PD-L1 expression in specific mRNA regions (−1,872/−1,862 base pairs) [31]. It is also promising for bladder cancer to employ the concurrent administration of anti-PD-L1 antibodies and STING pathway agonists, which counteracts immune evasion and significantly inhibits tumor progression. In our study, experiments demonstrated that ferroptosis-induced ICD synergizes with STING pathway activation to promote DC maturation and antigen presentation, while PD-1 blockade effectively reverses immunosuppressive microenvironments, drives cytotoxic T cell infiltration, and establishes long-term immune memory.

This study successfully constructed a mannose-modified multifunctional nano-delivery system that integrates ferroptosis induction, innate immune activation, and immune checkpoint blockade through a multi-mechanism synergistic strategy, providing an innovative therapeutic solution for bladder cancer [32]. The system employs TME-responsive design to achieve specific drug release in lymph nodes and tumor tissues, significantly enhancing antitumor immune responses [33]. Although this system has shown targeted advantages in the treatment of bladder cancer, their systemic toxicity and long-term effects still need to be systematically evaluated. Oxidative stress-sensitive nanoparticles may trigger complement bypass pathways. During perfusion therapy, nanoparticles may also be absorbed into the bloodstream through the bladder mucosa, which may exacerbate systemic toxicity.

Additionally, this strategy systematically inhibited tumor metastasis and recurrence through targeted modulation of immune activation status in TDLNs. Mannose receptor (MR) is a type C lectin receptor that is mainly expressed on tumor-associated macrophages (TAMs) displaying an M2-like phenotype [34]. Mannose-modified nanoparticles can be directly enriched into the core cell population of the immune response by targeting MR. It would be a great idea to perform flow cytometry analysis of cell surface MR expression and colocalization of the fluorescence signal of nanoparticles via fluorescent labels [35]. Furthermore, lymph node sections can be observed using confocal microscopy, and the distribution of nanoparticles in TAMs was clarified by fluorescently labeled nanoparticles colocalized with cell markers such as HMGB1 [36].

This work not only provides a theoretical basis for overcoming immunotherapy resistance but also establishes a technical foundation for developing combination immunotherapies based on microenvironment regulation. The mannose-modified nanodelivery system is significantly superior to single ICI or ferroptosis inducer therapy through precise targeting, multimodal therapy, and TME remodeling to achieve stronger inhibition of tumor growth and metastasis. ICI therapy improves the survival of patients with advanced bladder cancer, while many patients still develop immunotherapy resistance [37]. Notably, monotherapy groups (e.g., ​MPP@aPD-1/diABZI) showed limited metastasis inhibition despite partial increases in CD8^+^ T cell infiltration, attributable to the absence of ferroptosis-driven antigen release. In contrast, the combination group achieved near-complete metastasis suppression through tripartite mechanistic synergy: ​IKE-triggered antigen release, ​diABZI-mediated innate immune activation, and ​aPD-1-sustained T cell activity. These results underscore the necessity of spatiotemporally controlled drug delivery and multi-mechanistic integration, offering a breakthrough strategy for clinical metastasis therapy [38]. The exploration of in vivo transfer models will highlight the unique advantages of synergistic approaches over monotherapy. Future studies will focus on clinical translation to validate long-term efficacy and safety.

## Data Availability

The data used to support the findings of this study are available from the corresponding author upon request.
