# Supplementary material for: Nano-Immunotherapy Synergizing Ferroptosis and STING Activation in Metastatic Bladder Cancer
Source: Cyborg Bionic Syst. 2026 Jan 9;7:0458. doi: 10.34133/cbsystems.0458 (PMC12783508; doi:10.34133/cbsystems.0458)
Supplement: Supplementary 1 — Figs. S1 to S4 [file cbsystems.0458.f1.zip › Supplementary Materials.docx]

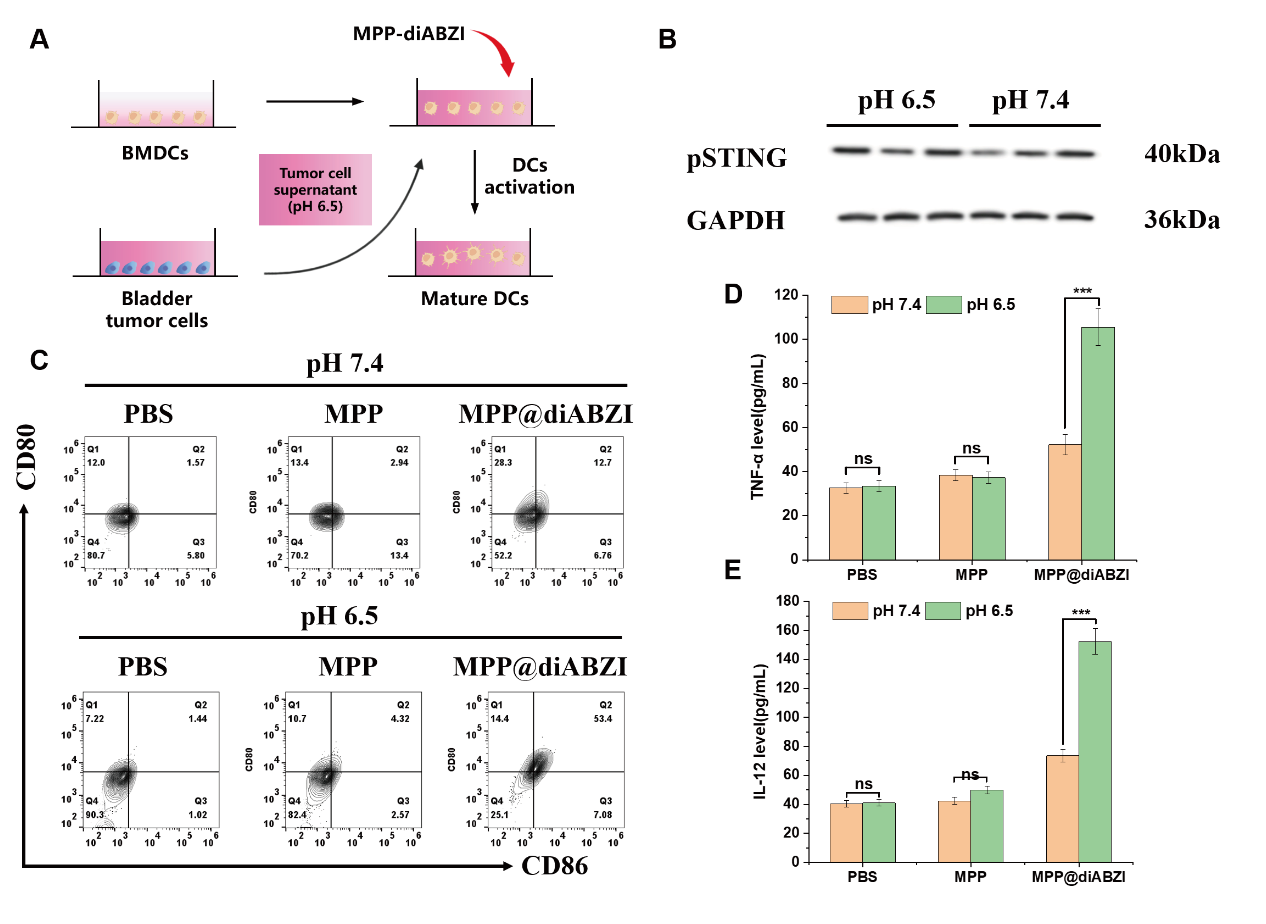


Figure S1. In vitro DCs activation by nanomedicine. (A) Schematic illustration of MPP@diABZI nanomedicine inducing in vitro DCs maturation. (B) The STING axis activation in BMDCs detected by WB. (C) The proportion of mature DCs in the BMDCs receiving nanomedicine treatments. (D) TNF-α and (E) IL-12 concentration in medium of BMDCs receiving different nanomedicine treatments detected by ELISA (n = 3). ***p < 0.001.


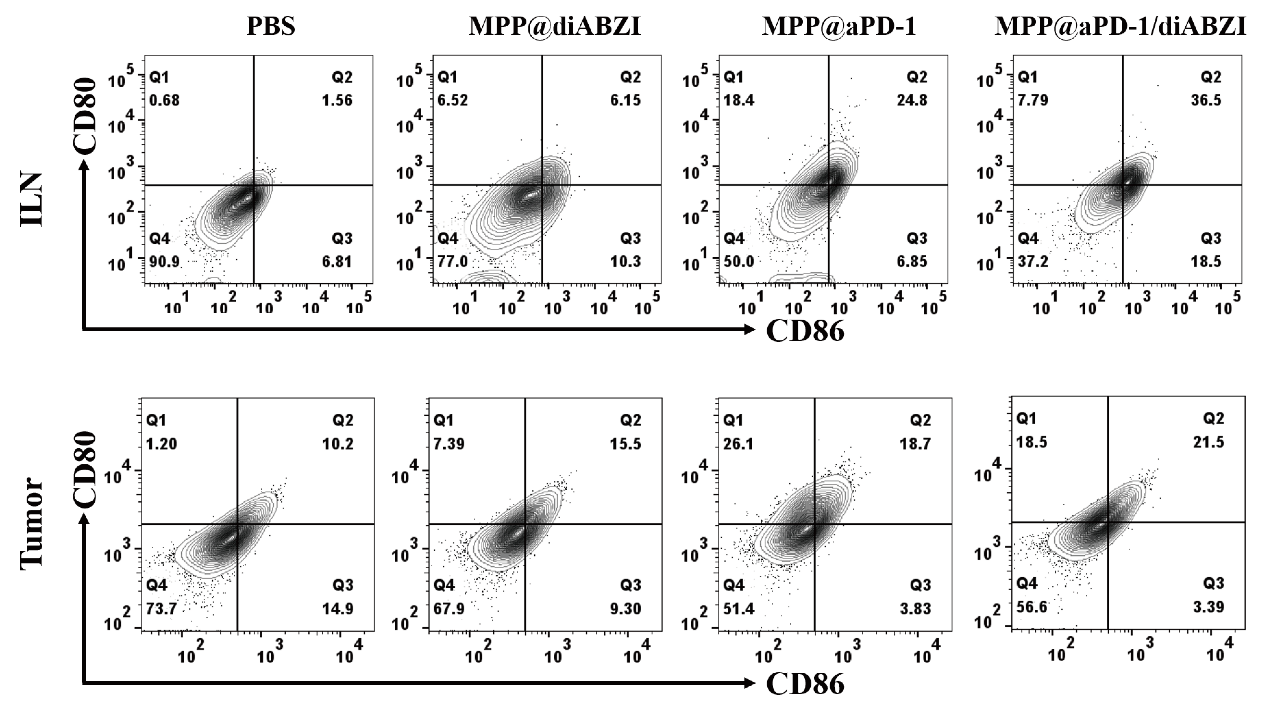


Figure S2. DCs and T cells activation in lymph nodes and tumor tissues analyzed by flow cytometry. The mature DCs in inguinal lymph nodes (ILN) and tumor tissues.


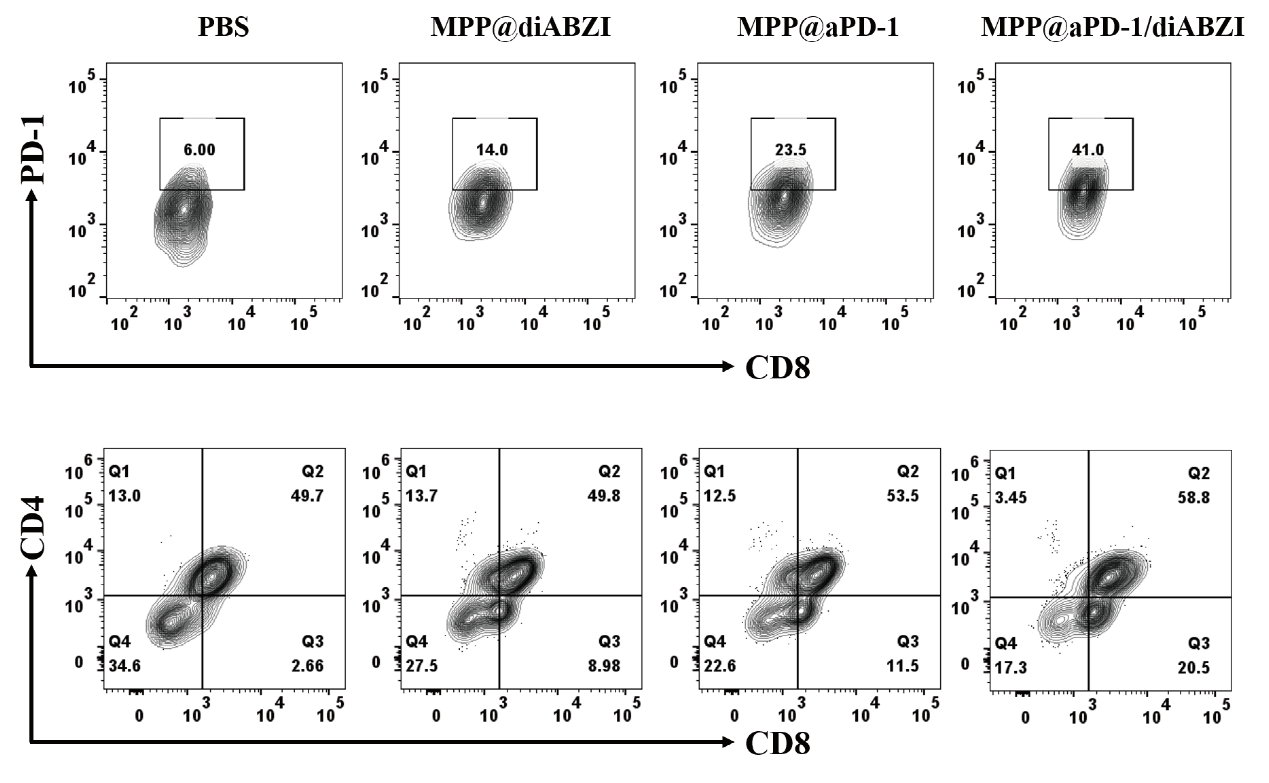


Figure S3. The PD-1+ T cells in inguinal lymph nodes (ILN) and CD8+ T cells in tumor tissues.


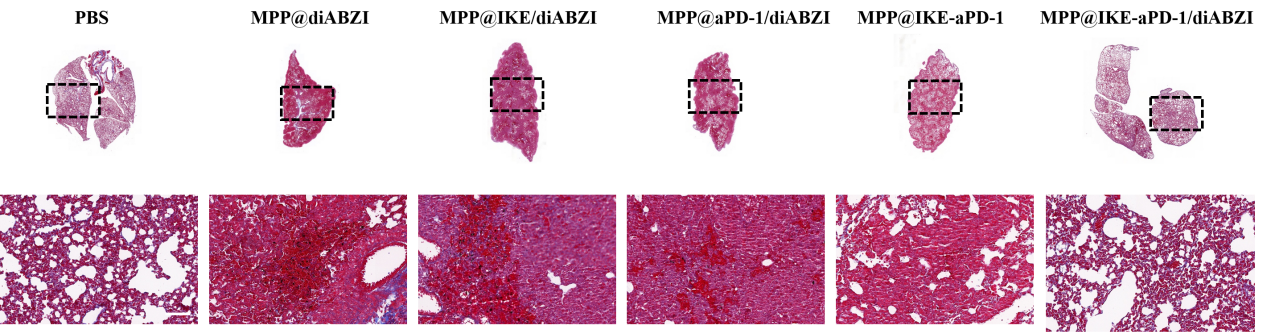


Figure S4. H&E staining images showing the tumor metastasis nodules in lung tissues.
