# Supplementary figures and images for: Nano-Immunotherapy Synergizing Ferroptosis and STING Activation in Metastatic Bladder Cancer
Source: Cyborg Bionic Syst. 2026 Jan 9;7:0458. doi: 10.34133/cbsystems.0458 (PMC12783508; doi:10.34133/cbsystems.0458)

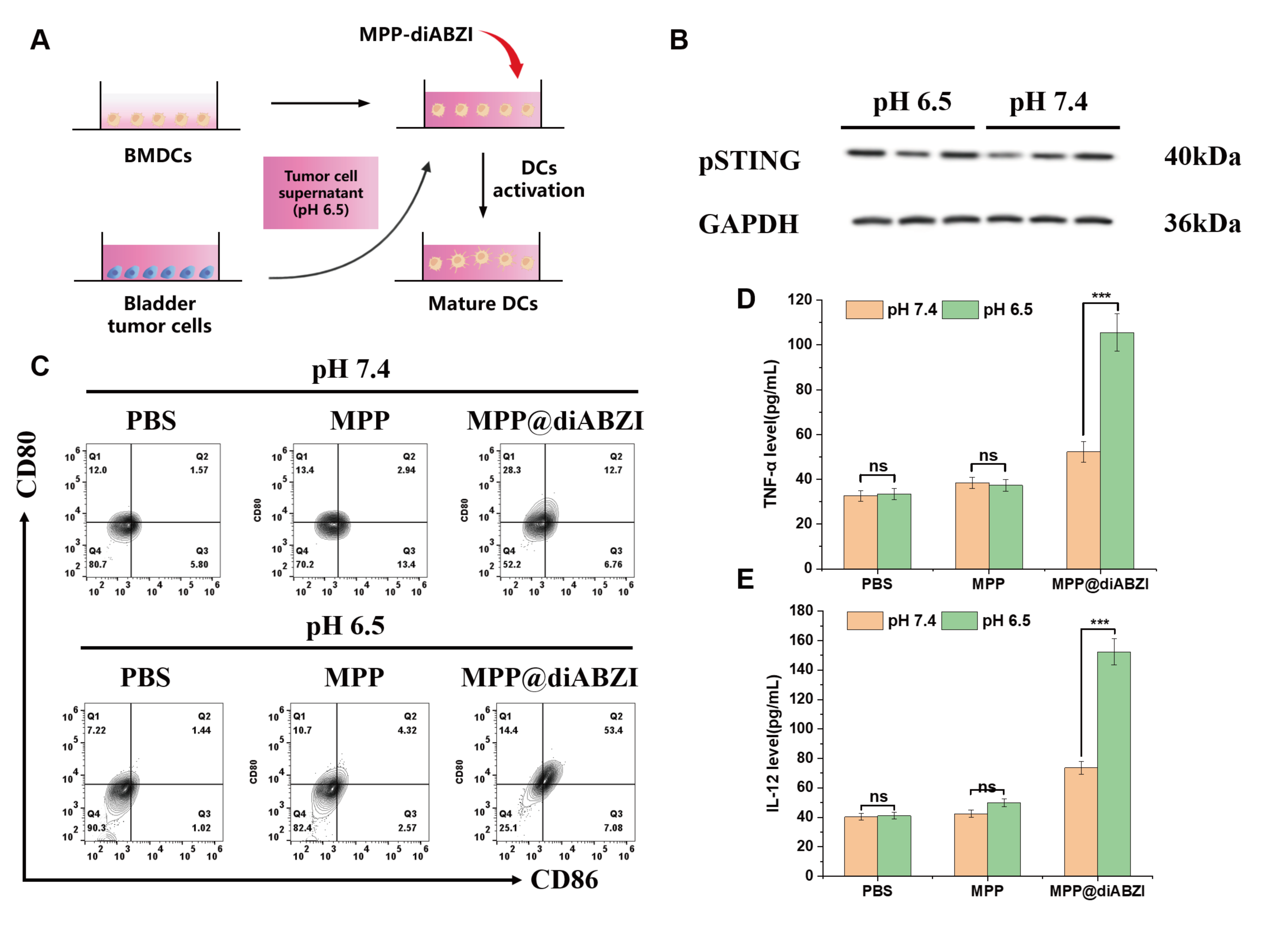

Supplement: Supplementary 1 — Figs. S1 to S4 [file cbsystems.0458.f1.zip › S1.tif]

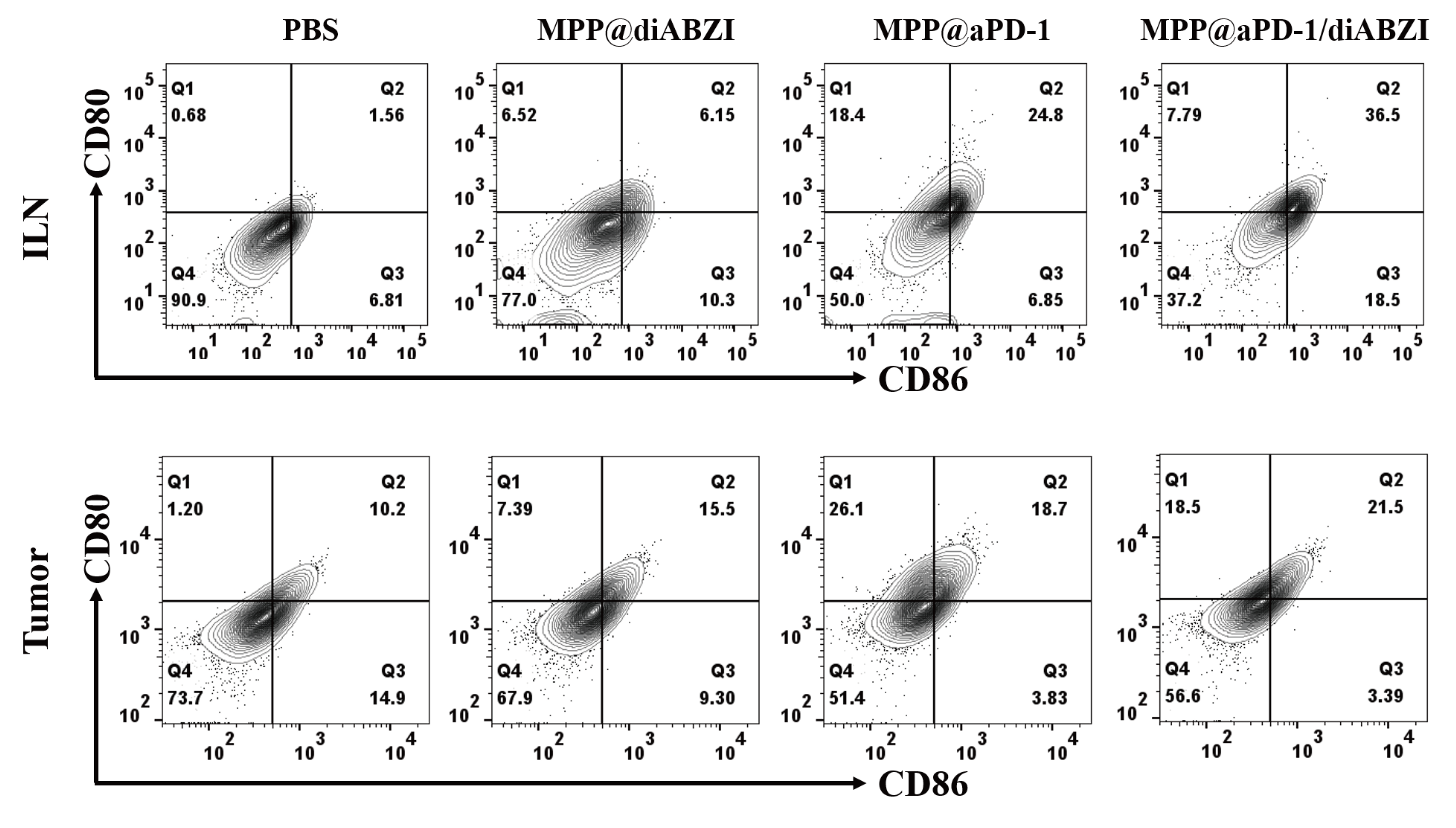

Supplement: Supplementary 1 — Figs. S1 to S4 [file cbsystems.0458.f1.zip › S2.tif]

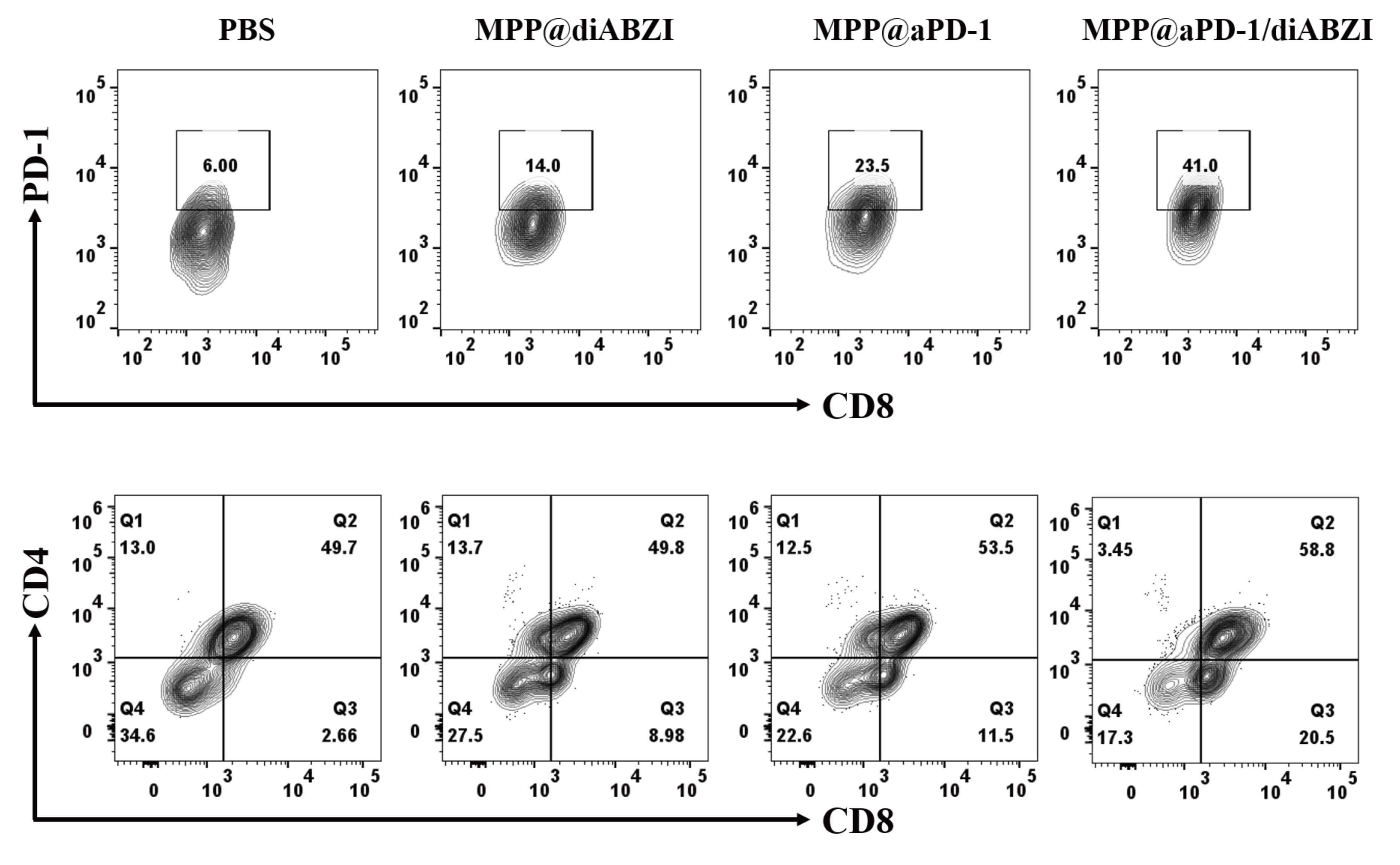

Supplement: Supplementary 1 — Figs. S1 to S4 [file cbsystems.0458.f1.zip › S3.tif]

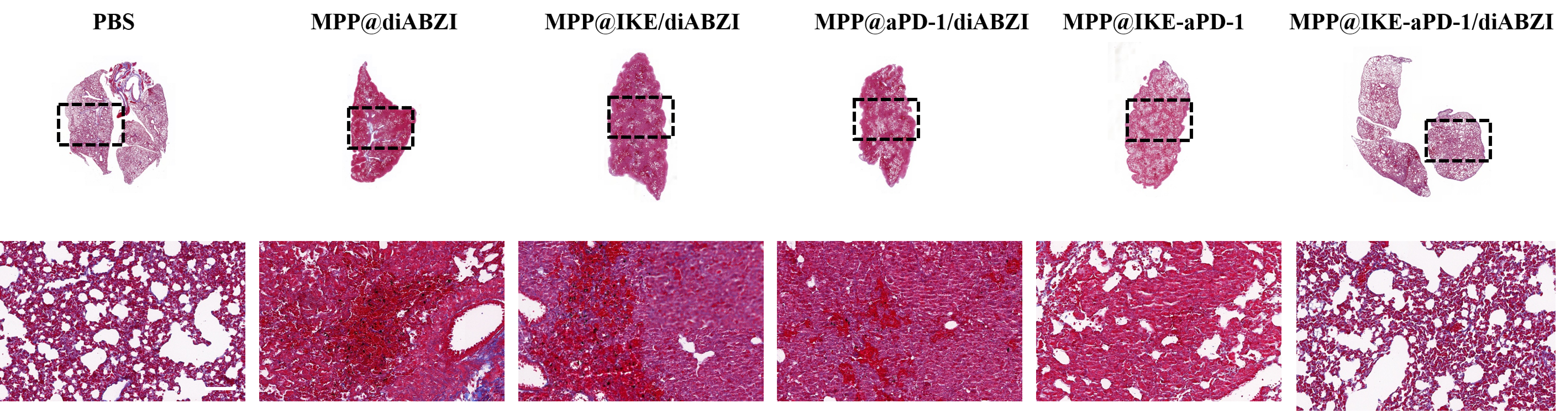

Supplement: Supplementary 1 — Figs. S1 to S4 [file cbsystems.0458.f1.zip › S4.tif]
